# Prenatal Glucocorticoid Exposure and Autism Spectrum Disorder: Current Evidence, Mechanisms, and Clinical Implications

**DOI:** 10.34763/jmotherandchild.20263001.d-26-00003

**Published:** 2026-07-18

**Authors:** Saim Mahmood Khan, Jawairya Muhammad Hussain, Yousif Daud Channa, Syed Zawiar Muhammad, Afnan Shaikh, Uzair Virk, Abdullah Aftab Khan, Muhammad Ali Shahzad, Surraiya Riaz Mahmood Khan

**Affiliations:** Karachi Medical and Dental College, Karachi, Pakistan; United Medical and Dental College, Karachi, Pakistan; Sohail University, Karachi, Pakistan

**Keywords:** Antenatal corticosteroids, Autism Spectrum Disorder, Glucocorticoids, Neurodevelopment, Pregnancy

## Abstract

Autism Spectrum Disorder (ASD) is a neurodevelopmental condition characterised by social difficulties and repetitive behaviours. Genetic and environmental factors play a key role in its aetiology. Antenatal glucocorticoids, widely used to improve outcomes in preterm infants, have raised questions about potential long-term neurodevelopmental effects. This review aims to summarise the current literature on the association between prenatal glucocorticoid exposure and neurodevelopmental outcomes, including ASD, explore proposed biological mechanisms, and discuss clinical and public health implications. A comprehensive literature search was conducted in PubMed, Web of Science and Google Scholar using relevant keywords, focusing on observational studies, meta-analyses, and clinical studies that addressed neurodevelopmental consequences of prenatal glucocorticoid exposure, with priority given to studies evaluating ASD risk. Glucocorticoids reduce neonatal respiratory complications in preterm infants. Long-term neurodevelopmental outcomes, including ASD, are uncertain: some observational studies suggest increased risk, especially in late-preterm or term infants, while randomised trials show minimal adverse effects. Potential mechanisms include epigenetic changes, HPA axis disruption, and altered hippocampal development. Glucocorticoids are essential for improving the survival of preterm infants. Current evidence does not establish a causal link with ASD, but potential long-term neurodevelopmental effects warrant cautious use, careful patient selection, and continued long-term follow-up. Further well-controlled studies using standardised neurodevelopmental assessments are needed.

## Graphical Abstract

**Figure:**
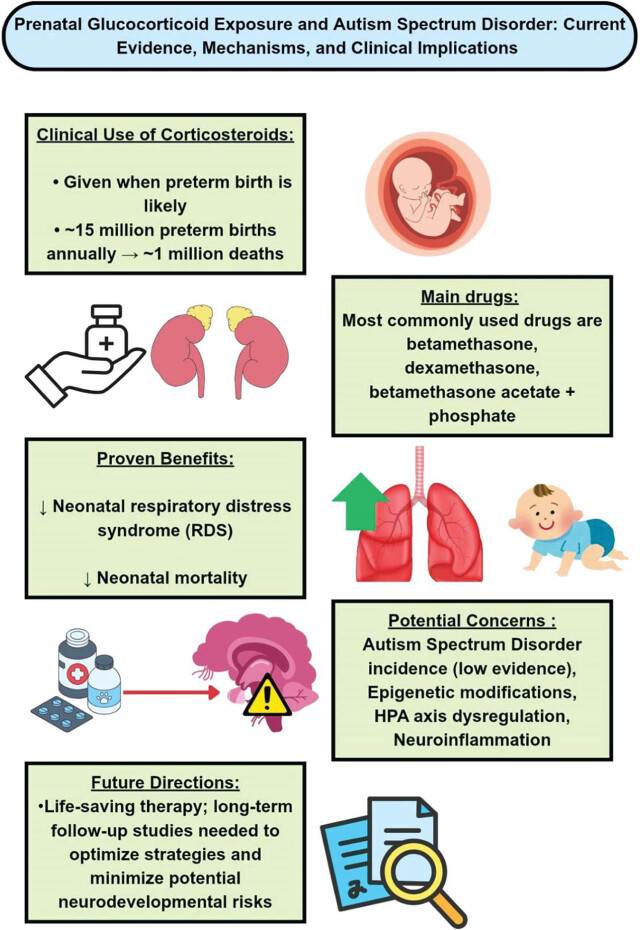
Prenatal glucocorticoids are vital for reducing neonatal respiratory complications in premature infants. However, emerging evidence suggests a potential association with increased risk of autism spectrum disorder (ASD). Proposed mechanisms include hypothalamic-pituitary-adrenal axis dysregulation, epigenetic modifications, and altered hippocampal development. While life-saving in preterm births, caution is advised in late preterm or term pregnancies. Further standardised studies are required to clarify long-term neurodevelopmental effects.

## 1. Introduction

Autism spectrum disorder (ASD) is a neurodevelopmental condition defined by persistent deficits in social communication and social interaction across multiple contexts. These challenges are typically accompanied by restricted and repetitive patterns of behaviour, interests, or activities. To meet diagnostic criteria, symptoms must present in early developmental periods and cause clinically significant functional impairment, provided they are not better explained by intellectual developmental disorder (intellectual disability) or global developmental delay (DSM-5-TR) [1]. While it is known that the appearance of symptoms related to ASD typically occurs between 12 to 18 months [2] of age, a clear diagnosis is possible when the individual is 24 months old [3]. A study by Salari et al. Presents a broader range of time by performing a meta-analysis that shows that the age of diagnosis is determined by the severity of the presenting symptoms, which range from 36 to 120 months, with an average of 55 months [4]. According to the Autism and Developmental Disabilities Monitoring Network, the estimated percentage of children with ASD for 2016 is 18.5 per 1,000 8-year-old children, with a male-to-female ratio of 4.3:1 [5]. Global data show considerable variation; however, direct comparisons should be made with caution due to differences in study periods, populations, and diagnostic criteria [6].

The aetiology of ASD involves a complex interplay of genetic, developmental, and environmental factors [7]. Beyond genetic risk, prenatal environmental exposures, particularly certain medications, can significantly impact neurodevelopment. Among prenatal exposures of interest, corticosteroid administration during pregnancy has received attention. Glucocorticoids (GCs) are prescribed for their immunosuppressive and anti-inflammatory properties, as well as for fetal lung maturation in high-risk pregnancies [8]. The clinical benefits of such an intervention have been well-established. A landmark randomised controlled trial by Gyamfi-Bannerman and colleagues found that administration of betamethasone (two 12 mg doses) to women at risk of late preterm delivery significantly reduces neonatal respiratory complications within 72 hours of birth [9]. Another randomised controlled trial by the ASTECS group found similar benefits in women undergoing elective cesarean section, with significantly reduced admissions to the NICU due to respiratory complications in the offspring of those who received antenatal steroids (RR 0.46; 95% CI 0.23–0.93) [10].

Despite these clear neonatal benefits, growing concerns surround the potential long-term neurodevelopmental consequences of antenatal corticosteroid (ACS) exposure. Some observational studies have reported associations between ACS exposure and subtle cognitive differences in early childhood [11]. However, these findings are not consistent across the literature, and large randomised trials and long-term follow-up studies have not demonstrated a clear association with major neurodevelopmental outcomes. This review therefore aims to critically appraise the current evidence on ACS exposure and neurodevelopment, with particular attention to studies exploring ASD. The goal is to synthesise biological hypotheses, clinical trial data, and epidemiological findings. We aim to highlight both the limitations of existing research and the need for further investigation.

## 2. Overview of antenatal corticosteroid use

Globally, around 15 million babies are born each year prematurely, resulting in over a million neonatal deaths [12]. In one of the earliest landmark studies, a randomised, placebo-controlled trial of ACS was conducted among 282 pregnant women at risk of preterm birth, based on the hypothesis that ACS could reduce the high mortality rates among preterm neonates [13]. Since then, robust evidence has confirmed these life-saving benefits of ACS. A Cochrane review estimates that ACS therapy significantly reduces the risk of perinatal death, neonatal death, and respiratory distress syndrome (RDS) [14]. The three most used drugs for ACS therapy are dexamethasone phosphate (Dex-p), betamethasone phosphate (Beta-p), and a combination of betamethasone acetate (Beta-ac) with betamethasone phosphate (Beta-p) [15]. Due to its affordability and availability, the World Health Organisation (WHO) currently recommends Dex-p, administered as four doses of 6 mg intramuscularly (total 24 mg) [16] at 12-hour intervals; this specific dosing regimen produces more sustained plasma levels and avoids the sharp peaks seen with betamethasone [17]. Although the oral bioavailability of betamethasone and dexamethasone is similar, intramuscular administration remains the standard clinical route [18].

Betamethasone is typically administered as a combination of Beta-p and betamethasone acetate (Beta-ac), with the phosphate ester hydrolysing rapidly to produce a quicker systemic rise, while the acetate ester hydrolyses more slowly, prolonging activity [13]. Betamethasone and dexamethasone are fluorinated corticosteroids with a greater ability to cross the placenta compared to non-fluorinated corticosteroids [19]. From a pharmacokinetic perspective, it is known that betamethasone is metabolised by CYP3A4 in adults and CYP3A7 in the feto-placental unit [20], with other enzymes such as 11β-hydroxysteroid dehydrogenase (11β-HSD) types 1 and 2 playing a role in the formation of active metabolites [21]. Levels of betamethasone are maximal in the mother's serum after 1 hour, with maximal levels being seen in the fetus after 1-2 hours. Its half-life is 6 hours in the mother and 12 hours in the fetus, with levels being undetectable 48 hours after the second injection [22].

Studies show no significant difference in mortality between dexamethasone and betamethasone, as the confidence interval (CI) spans both potential benefit and harm (Response Rate (RR): 1.03, 95% CI 0.66–1.63; 5 trials, 2105 infants; moderate-certainty evidence). Similarly, the risk of RDS may not differ significantly (RR 1.06, 95% CI 0.91–1.22; 5 studies, 2105 newborns; high-certainty evidence). The effect on intraventricular haemorrhage also appears minimal [23]. For this reason, betamethasone formulations are often combined to optimise efficacy and minimise the number of maternal injections [24]. Because both dexamethasone and betamethasone cross the placenta in active form, they may influence the maturation of the fetal hypothalamic–pituitary–adrenal (HPA) axis. Such alterations could potentially disrupt neurodevelopmental programming, a pathway increasingly implicated in the aetiology of ASD.

Animal studies suggest that ACS exposure at clinical doses can alter HPA regulation and, in some cases, neurodevelopmental programming [25]. Other organ systems may also be affected in both preterm and full-term animals [24]. However, extrapolating these findings to humans remains challenging. A population-based cohort study from Ontario, Canada, reported that term-born infants exposed to ACS had an increased risk of suspected neurocognitive disorders (adjusted hazard ratios (aHR) 1.12, 95% CI 1.05–1.20). These children also had a greater likelihood of undergoing audiometry (aHR 1.20, 95% CI 1.10–1.31) and visual testing (aHR 1.06, 95% CI 1.01–1.11) by the age of five [26]. While the study did not specifically evaluate ASD, it illustrates ongoing debate about potential long-term effects. Importantly, other large randomised and follow-up studies have not consistently demonstrated adverse neurodevelopmental outcomes, highlighting the uncertainty in this field.

Thus, while ACS therapy remains an undeniably essential intervention for improving neonatal survival and reducing respiratory morbidity in preterm births, questions about possible long-term effects, including neurodevelopmental outcomes, continue to warrant careful study.

## 3. Neurodevelopmental programming and corticosteroids

The concept of Fetal programming, also known as the Developmental Origins of Health and Disease, refers to the concept that exposures during critical periods of development can permanently influence organ structure, function, and metabolism, including the brain. Mechanisms include epigenetic modifications, altered hormone signalling, and organ adaptation, which may predispose individuals to neurodevelopmental, psychiatric, and cardiometabolic disorders [27].

### 3.1 Observational studies

Large population-based observational studies have compared neurodevelopmental outcomes in children exposed versus unexposed to ACS [15]. Interpretation of these findings is challenging, since ACS is usually administered in pregnancies already complicated by conditions such as hypertension or preterm rupture of membranes, which themselves carry increased risks. Most observational datasets also lack detailed clinical information, which means it is challenging to distinguish the effects of the drug from those of the underlying complications [21].

### 3.2 Meta-analysis

A recent meta-analysis, which included RCTs and quasi-experimental studies with offspring followed up from age 1 up to ~18 years, found that 19 of 23 neurodevelopmental outcomes showed no association with ACS exposure. Among the more long-term outcomes (school-age, academic performance), results were also generally null or showed only modest differences in nonverbal intelligence or visual memory. These findings strengthen the evidence that any risk signals are small and uncertain, even in longer follow-ups [28]. Similarly, a meta-analysis of 30 studies involving >1.25 million children reported that a single ACS course significantly reduced neurodevelopmental impairment among extremely preterm infants (aOR, 0.69; 95% CI, 0.57–0.84). In contrast, late-preterm infants showed a modest increase in neurocognitive disorders (aHR, 1.12; 95% CI, 1.05–1.20), while term infants were associated with higher risks of mental/behavioral disorders (aHR, 1.47; 95% CI, 1.36–1.60) and neurocognitive disorders (aHR, 1.16; 95% CI, 1.10–1.21). This data suggests that potential outcomes may vary significantly depending on the gestational age at exposure [29]. Across multiple neurodevelopmental domains, no consistent trends of elevated risk have been identified with ACS exposure.

### 3.3 Development timing and critical windows

The timing of these critical developmental windows may help explain these associations.

In the brainstem and spinal cord, CNS neurogenesis begins at 4 post-conception weeks (6 weeks of gestation) [30]. Neocortical plate synaptogenesis starts as early as 18 post-conception weeks (20 weeks of gestation) [31], with peak synaptic density in the prefrontal cortex reached around 15 months after birth—much later than in sensory cortices. Because these developmental windows are so prolonged, early perturbations, including ACS exposure, have the potential to cause lasting effects on brain architecture [32].

### 3.4 Animal studies

Animal studies provide mechanistic insights into how ACS may alter neuronal development. In mice, a single antenatal dexamethasone dose (0.4 mg/kg at embryonic day 15.5) reduced hippocampal volume, proliferation, and body weight during development, although some changes were transient [33]. Broader reviews of animal models indicate reduced neuronal density, altered serotonin and dopamine signalling, and persistent behavioural changes following ACS treatment [26]. While such programming effects could contribute to later cognitive or behavioural vulnerabilities, the specific relationship between ACS and ASD remains uncertain and requires further targeted study. Animal models provide us with great biological clues, but we need human data to know for sure.

In summary, the results from the various study designs have been inconsistent but also reassuring, demonstrating no long-term neurodevelopmental effects in the randomised studies, and the meta-analyses demonstrating significant benefits to extremely preterm children, with only modest effects in the later gestational ages. These programming effects, while they may play a role in the cognitive and behavioural difficulties that can also be seen, have an unclear relationship to ACS and ASD, and more study is needed to understand the relationship between the two conditions. Animal models provide us with great biological clues, but we need human data to know for sure.

The next section will discuss the implications of the epidemiological data for the risk of ASD.

## 4. Evidence linking prenatal corticosteroids to ASD Risk

### 4.1 ASD-specific evidence

#### 4.1.1 Large population-based cohort studies

Current epidemiologic evidence suggests a possible association between prenatal GC exposure and ASD risk.

In a large cohort, children born to mothers with comparable underlying conditions showed higher adjusted rates of ASD following prenatal GC exposure during pregnancy compared with unexposed peers (6.6% vs. 4.3%; RR 1.5, 95% CI, 1.2–1.9). Similarly, among offspring of mothers with autoimmune or inflammatory disorders, ASD risk was 4.8% in the exposed group versus 3.8% in the unexposed group (RR 1.3, 95% CI 1.1–1.5). Although these findings suggest a link, residual confounding remains a concern, as maternal disease itself may increase risk. To address this, the study adjusted for multiple parental and perinatal factors, including demographics, smoking, socioeconomic status, psychiatric and autoimmune disorders, and concurrent medications. For pregnancies at risk of preterm delivery, further adjustments typically include gestational diabetes, preeclampsia, and maternal infections [34].

A large Finnish national cohort of 670,097 children, including 14,868 (2.2%) with prenatal corticosteroid exposure, reported a cumulative incidence of any mental or behavioural disorder of 12.01% in exposed versus 6.45% in non-exposed children (absolute difference, 5.56% [95% CI, 5.04–6.19]; P < .001). Multivariable models confirmed this association (HR, 1.33 [95% CI, 1.26–1.41]; P < .001). Subgroup analyses indicated a stronger association among term-born children (incidence, 8.89% vs. 6.31%; HR, 1.47 [95% CI, 1.36–1.69]), while the adjusted association was not statistically significant in preterm infants. Importantly, sibling-comparison analyses yielded similar findings, suggesting that shared familial factors were unlikely to account for the observed link [35].

#### 4.1.2 Smaller mechanistic / biomarker studies

Beyond large epidemiological cohorts, smaller studies have examined maternal cortisol regulation as a potential mechanism. In one study of 84 mother–child pairs, lower prenatal maternal cortisol levels were significantly associated with higher ASD symptom scores in male offspring (slope −0.45, t = −2.48, p = .01), but no association was observed in females (slope 0.10, t = 0.71, p = .48). These analyses were carefully adjusted for gestational age, obstetric risk factors, socioeconomic status, child's sex, age, and birth order [36]. Taken together, these findings provide preliminary evidence of sex-specific effects, though sample sizes remain small and causality cannot be established. While most studies directly evaluating ASD outcomes remain inconclusive, several large-scale human cohort studies provide quantitative evidence of broader neurodevelopmental and behavioural effects following ACS exposure.

### 4.2. Neurodevelopmental and psychiatric outcomes

#### 4.2.1 Randomised controlled trial follow-up data

Longitudinal follow-up of clinical trials provides a more nuanced picture. Two trials following children at 2 years of age after repeated GC courses found no significant differences in physical or brain development, and another study in younger children reported similar results. However, one trial observed a non-significant increase in cerebral palsy risk after serial ACS courses (RR 5.7, 95% CI 0.7–46.7; p = .12), raising questions that require further investigation. Concerns about adverse brain effects have been most pronounced with multiple courses or late-preterm exposure, while single-course treatment before 34 weeks is generally associated with survival and pulmonary benefits [37].

Long-term neurocognitive data for late-preterm exposure are limited to the original GCs trial, in which participants were randomised between 24 0/7 and 35 6/7 weeks. In the 30-year follow-up, exposure between 30.9 and 34.6 weeks, with a median delivery at 35 weeks (range 33.4–38.0), showed no differences in intelligence, working memory, attention, or other cognitive measures; 34% (n = 66) of this cohort delivered at term. Importantly, GCs remain safe in maternal critical illness, including sepsis [38]. Nevertheless, Current evidence on long-term outcomes, including the risk of ASD, is still limited and needs further research. Current guidelines recommend a single repeat course for women at risk of preterm delivery while emphasising the need for continued surveillance of long-term outcomes [39]. Human studies on prenatal corticosteroid exposure and ASD/Neurodevelopmental outcomes are illustrated in Table 1.

**Table 1.** Summary of key human studies on Prenatal Corticosteroid exposure and ASD/Neurodevelopmental outcomes.

**Author**	**Year**	**Study population**	**Study design**	**Number of cases**	**Outcome**	**Corticosteroid**	**Main Findings**
Räikkönen K et al. [33]	2020	Finnish Institute for Health and Welfare	Population-based retrospective cohort study	n = 670 097 (Total) n = 14 868 (exposed) n = 655 229 (unexposed)	Any childhood mental and behavioural disorder	Betamethasone	Antenatal corticosteroid treatment was significantly linked to an increased risk of mental and behavioural disorders in children
Laugesen K et al. [34]	2025	Danish medical birth registry	Population-based cohort study	n = 1 061 548 (Total) n = 31 518 (born to mothers at risk of preterm delivery) n = 288 747 (born to mothers with autoimmune or inflammatory disorder) n = 741 283 (General population)	Mental disorders in offspring	Prednisolone Prednisone Betamethasone Dexamethasone Hydrocortisone Triamcinolone Methylprednisolone	Prenatal exposure to systemic glucocorticoids was significantly associated with a greater risk of specific mental disorders
Bannerman CG et al. [8]	2016	Eunice Kennedy Shriver National Institute of Child Health and Human Development (NICHD)	Multicenter, randomised trial	n = 2831 (total) n = 1429 (exposed) n = 1402 (unexposed)	Early respiratory support or death within 72 hours after birth.	Betamethasone	Antenatal administration of betamethasone to women at risk of late preterm delivery was associated with a significant reduction in respiratory complications among newborns
Ninan K et al. [29]	2022	Systematic review and meta-analysis focusing on long-term outcomes of antenatal corticosteroid exposure	Systematic review and meta-analysis of randomised clinical trials (RCTs), quasi-RCTs, and cohort studies	n = 30 studies (total) n = 10 studies (single course vs non-exposure) n = 20 studies (unknown number of courses vs non exposure)	Primary outcome: Any adverse neurodevelopmental/psychological disorders secondary outcomes: psychological, developmental, growth, metabolic, and cardiorespiratory measures.	Betamethasone Dexamethasone	A single course of antenatal corticosteroids was linked to reduced neurodevelopmental impairment in extremely preterm children but increased risk of adverse neurocognitive or psychological outcomes in late-preterm and full-term children

#### 4.2.2 Meta-analyses and broader neurodevelopmental outcomes

Complementing the trial data, a meta-analysis of nearly 9,000 preterm small-for-gestational-age infants demonstrated that ACS reduced neonatal mortality (12.8% vs. 15.1%; pooled OR 0.63, 95% CI 0.46–0.86), though reductions in morbidity outcomes were inconsistent [40]. More recently, a large Chinese multicenter cohort of very preterm infants (n = 2,514) reported that complete ACS exposure was associated with modestly faster postnatal weight gain (median 14.6 g/kg/day) and lower odds of extrauterine growth restriction (adjusted OR 0.60–0.64) compared with non-exposed infants [41].

Taken together, these findings indicate that ACS can have measurable long-term associations with neurodevelopment, behaviour, and growth outcomes in humans. However, interpreting these results requires caution because none of these studies specifically examined ASD diagnosis, and important confounders such as gestational age, maternal health, and postnatal environment limit causal inference. Randomised trials and long-term follow-up studies have generally been reassuring, without clear increases in major neurocognitive impairment. Conversely, some large observational studies have reported modest associations with ASD or broader behavioural diagnoses, particularly among term infants, although these findings may be influenced by underlying maternal conditions and differences in diagnostic practices. Smaller mechanistic studies point to possible effects on stress regulation and potential sex-specific patterns, but these results are still preliminary. Thus, while human evidence underscores the need for ongoing follow-up, the direct causal relationship between ACS and ASD remains unproven.

Thus, the overall available evidence supports the notion that prenatal GCs could be related to ASD, although the quality of the evidence is limited by confounding factors. More robust associations have been found for general mental and behavioral outcomes, especially in term or late preterm babies. The results obtained so far underscore the importance of the gestational period and patterns of GCs, but also the need for long-term studies specifically focused on ASD.

## 5. Epigenetic, neuroinflammatory, and endocrine mechanisms of corticosteroid exposure

Epigenetic mechanisms provide a key pathway by which prenatal corticosteroid exposure may influence neurodevelopment. Unlike mutations, which permanently alter DNA sequences, epigenetic changes regulate gene expression through reversible modifications such as DNA methylation and histone remodelling. Corticosteroids may also affect DNA methyltransferases as well as histone deacetylases, which in turn affect transcriptional activities [42,43,44]. Once they bind to the glucocorticoid receptors in the cytoplasm, corticosteroids form a complex that, upon translocation into the nucleus, binds to glucocorticoid response elements, resulting in altered gene expression [45]. These actions may be important for long-term effects on brain development as well as behavioural outcomes.

Gestational age appears to modify these effects. In a prospective observational study on children born near term (median 37+6 weeks), antenatal betamethasone exposure for threatened pre-term birth has been associated with lower IQ scores and slight increases in Attention-deficit/hyperactivity disorder (ADHD) symptomatology at 8-9 years of age, with no alterations detected in HPA axis activity [46]. The above study is particularly pertinent as there is a known genetic link between ADHD and ASD, as exemplified by overlapping susceptibility genes such as SHANK2. The SHANK2 gene is a crucial postsynaptic scaffolding gene that is essential for the organisation of excitatory synapses and for neuronal communication. The SHANK3 gene is another crucial scaffolding gene that is essential for postsynaptic density and is involved in the formation, maturation, and communication of synapses. Genes such as SHANK2 and SHANK3 are not classical regions such as 11q15.5 or 15q11-13 that are typically subject to classical imprinting. Instead, they are subject to epigenetic modification. Consequently, alterations in SHANK2 and SHANK3 are both associated with increased ASD susceptibility. Findings like this raise the possibility of overlapping mechanistic pathways [47]. Such findings reinforce the necessity to screen children with one condition for features of the other. Further evidence comes from prenatal corticosteroid treatment, which affects infant stress regulation; infants exposed to corticosteroids show exaggerated cortisol levels between 6 to 12 months of age, which is a critical period for socio-emotional development [48].

Beyond epigenetics, neuroinflammation further links corticosteroid exposure to atypical development. The interaction of GCs and stress may result in hippocampal neuroinflammation, which may cause disturbances in the structure and function of the hippocampus and result in epilepsy, depression, and cognitive impairment [49]. The abnormal development of the hippocampus and its connections has been proven to influence the symptoms of ASD because the maturation of the hippocampus corresponds with the age when the symptoms of ASD appear [50]. This is an important mechanism in understanding the role of neuroinflammation caused by the exposure of corticosteroids in the pathogenesis of ASD. The exposure of the body to excessive cortisol also results in the HPA axis's hyperactivation and the inflammatory response, which are the risk factors for major depressive disorders [51]. The frequent co-occurrence of depression and ASD highlights how corticosteroid exposure may influence overlapping neuropsychiatric trajectories.

**Figure 1.**
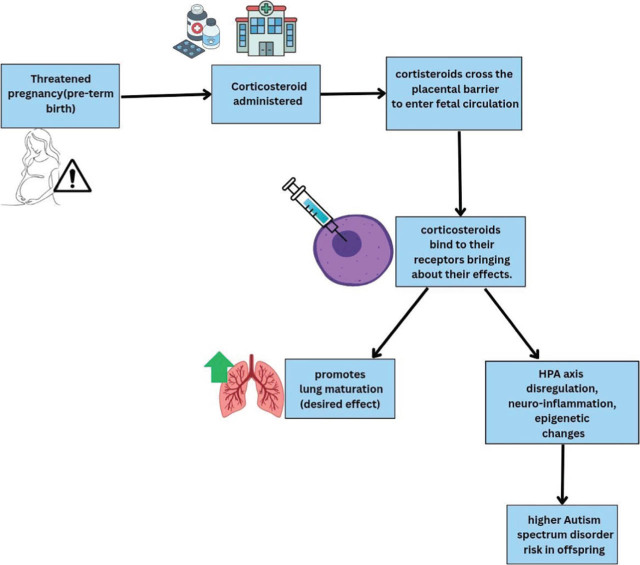
Conceptual Framework – Pathway from Antenatal Corticosteroid Exposure to ASD Risk.

Lastly, it appears that sex-specific susceptibility also affects outcomes, as evidenced by epidemiological studies that consistently demonstrate a higher prevalence of ASDs in males, although the biological basis for this phenomenon is not fully understood [52]. This indicates that the sex of the fetus could influence outcomes following exposure to glucocorticoids. This observation suggests that fetal sex may interact with glucocorticoid exposure to modulate neurodevelopmental risk. Mechanisms linking prenatal corticosteroid exposure to ASD are summarised in Figure 1, which outlines the proposed biological pathway, and detailed in Table 2, which compiles specific epigenetic, neuroinflammatory, and endocrine mechanisms supported by current evidence. These cellular mechanisms show how the medication directly affects the fetal brain. However, a mother's natural stress hormones also play a major role in this process. We now need to look at how these natural hormones interact with the prescribed steroids during pregnancy.

**Table 2.** Proposed Mechanisms Linking Prenatal Corticosteroid Exposure to ASD.

**Biological mechanism**	**Description**	**Evidence**	**Potential effect**
HPA axis dysregulation	Hormonal imbalance, which might increase cortisol levels in fetus	Elevated prenatal maternal cortisol programs the infant HPA axis, leading to larger cortisol responses to stress at 6 and 12 months of age [48].	HPA axis dysregulation is associated with increased risk of depression and has been implicated in ASD pathogenesis.
Epigenetics	Corticosteroids interact with enzymes that control epigenetic processes.	Glucocorticoids interact with DNA methyltransferases and histone deacetylases, modifying transcriptional activity without altering DNA sequence [44].	Epigenetic changes can persist across the lifespan and may contribute to transgenerational ASD risk.
Neuro inflammation	Excessive cortisol hyperactivates the HPA axis, leading to neuro inflammation	Glucocorticoids can mediate hippocampal inflammation; hippocampal abnormalities are consistently implicated in ASD [50].	Hippocampal inflammation is linked to depression and cognitive impairment, conditions that frequently co-occur with ASD.
Sex-specific vulnerability	Fetal sex may modulate the neurodevelopmental effects of glucocorticoid exposure	Steroid-related biomarkers in maternal serum differ by sex and are associated with altered autism risk [52].	May help explain the consistently higher prevalence of ASD in males.

ASD=Autism Spectrum Disorder.

HPA-axis=Hypothalamic–pituitary–adrenal axis.

## 6. Role of maternal stress and endogenous glucocorticoids

Maternal stress during pregnancy is a well-recognised factor shaping both maternal physiology and fetal development. One study utilising the Perceived Stress Scale reported a mean score of 25.6 among pregnant participants, indicating moderate psychological stress [53]. Physiologically, pregnancy is accompanied by placental corticotropin-releasing hormone (CRH)–driven increases in cortisol. Exogenous corticosteroid administration, particularly at higher doses (e.g., 0.25–0.5 mg of dexamethasone), can suppress endogenous cortisol production and disrupt HPA axis activity [54]. Such disruptions are thought to interact with maternal stress pathways, although long-term consequences for child neurodevelopment remain uncertain.

Experimental and clinical data indicate that corticosteroids have a variable influence on hippocampal and amygdalar activity that resembles but does not exactly parallel that of endogenous cortisol [55]. Pregnancy itself causes adaptive changes in the HPA axis; the fetoplacental unit is a key contributor to high cortisol levels that are necessary for a healthy outcome [56]. Some data indicate that exogenous corticosteroids could influence fetal development, including fronto-parietal areas of the brain that are involved in cognitive and behavioural processes [57], although other data, including long-term follow-up of randomised controlled trials, have failed to demonstrate this. This discrepancy highlights the need for caution when extrapolating mechanistic findings to clinical risk.

Corticosteroid therapy also has effects on fetal blood supply. A longitudinal study found out that betamethasone administration leads to a lower pulsation index in the umbilical artery [58], which can increase susceptibility to fetal hypoxia. Excessive exposure has also been linked in some observational studies to congenital cardiovascular anomalies [59], which themselves may carry a higher risk of ASD [60]. However, causal interpretation is limited, as many of these associations are confounded by underlying obstetric risk factors.

Biomarker research also sheds further light on stress and steroid hormone pathways [61]. Cortisol/cortisone ratios in urine or hair have been used as a biomarker of stress and maternal vulnerability [62]. HPA axis abnormalities during pregnancy may reduce placental 11βHSD2 activity, which normally protects the fetus from the adverse effects of maternal cortisol by converting cortisol to cortisone [63]. Reduced activity may increase exposure of the fetus to cortisol, especially in males, which may partly explain the sexual dimorphism in epidemiological studies [63]. However, the data are not clear-cut, and some studies failed to show sexual differences.

Thus, maternal stress and exogenous corticosteroids appear to interact through a common endocrine pathway, with putative biological mechanisms underlying the relationship with neurodevelopmental outcome. However, the long-term clinical data are also inconsistent, with several studies failing to show significant neurocognitive impairments.

## 7. Comparative discussion: synthetic vs. endogenous glucocorticoids

GCs were named as such to highlight their role in the regulation of glucose metabolism, their site of synthesis in the adrenal cortex, and their steroid chemical structure. Endogenous GCs are those produced by the human body, i.e., in the adrenal cortex, and include cortisol and its inactive metabolite, cortisone [64]. Synthetic GCs are a class of pharmacological agents that bind to the same GR as endogenous GCs but differ in their pharmacodynamics. These include betamethasone, dexamethasone, prednisone, and methylprednisolone [65]. Their clinical indications in pregnancy have already been described above.

All GCs act by binding to the GR, normally located in the cytoplasm, complexed with chaperone proteins. After binding, the GR undergoes conformational change and translocates to the nucleus, where it acts in collaboration with glucocorticoid response elements to carry out activation or repression of transcription [66]. The anti-inflammatory actions of GCs result from transcriptional repression, whereas the adverse effects of high or prolonged doses result from transcriptional activation [67]. GCs have been shown to have non-genomic actions, including lipid membrane changes, alteration of the Mitogen-Activated Protein Kinase cascade, and the regulation of mitochondrial gene transcription [68]. However, the differences in these actions between endogenous and synthetic GCs are yet to be sufficiently described, with few studies offering a direct comparison [69]. Synthetic GCs such as dexamethasone show a greater potency than cortisol.

Another critical distinction is observed in their selectivity to the mineralocorticoid receptors (MR) involved in the body's salt-water balance. While endogenous GR binds to both GR and MR, most synthetic agents are GR-selective, with their MR activity only being observed at higher doses. This selectivity reduces mineralocorticoid-related adverse effects such as hypertension and oedema at their therapeutic doses [70]. Endogenous GCs are classified as short-acting, with biological half-lives under 12 hours. In contrast, synthetic compounds display extended half-lives that vary by agent: prednisolone and methylprednisolone are intermediate-acting (18–36 h), whereas dexamethasone and betamethasone are long-acting (36–54 h). This is partly due to their resistance to metabolism in the liver and a slower systemic clearance, while cortisol undergoes rapid inactivation and excretion [71]. The longer duration of synthetic corticosteroids enhances therapeutic efficacy and allows for a longer dosing interval.

During pregnancy, these pharmacological differences between endogenous and synthetic GCs are accentuated by placental physiology. GCs readily diffuse across the placenta due to their lipophilic nature. However, the placental enzyme 11βHSD type 2 inactivates the endogenous cortisol to cortisone, thereby limiting fetal exposure. On the other hand, synthetic GCs resist this enzymatic barrier and can cross freely into the fetal circulation. In addition to their higher potency and longer half-lives, this resistance to 11βHSD type 2 contributes to synthetic corticosteroids leaving more pronounced effects on fetal development compared with endogenous cortisol [72]. While synthetic GCs also differ from endogenous GCs by their higher affinity for GR than MR—potentially influencing neurodevelopmental pathways- there is currently no direct evidence linking this property to ASD risk. Rather, it should be considered a hypothesis requiring further investigation.

## 8. Ethical and public health considerations

The benefits of ACS are well-documented and extend to the mother and fetus, especially in the prevention of mortality and respiratory distress in preterm births [29]. Nevertheless, the long-term neurodevelopmental consequences of ACS, such as the increased risk of ASD and related conditions, are a source of concern. In terms of applying bioethical principles, autonomy and beneficence are the most relevant in the context of the present discussion. Therefore, the mother should be well-informed about the benefits of the procedure and the limitations of the available information. Evidence supports the fact that the open communication of information is likely to result in increased patient trust and promote the autonomy of the patient in the treatment regimen [73]. Such an approach, in addition to the open discussion of the adverse effects of the treatment regimen, is likely to result in an informed decision by the patient and subsequently increase patient satisfaction.

The clinical benefits of ACS also vary significantly by gestational age, which influences the ethical balance. For extremely preterm infants (23–25 weeks), ACS use can increase survival by up to 15–18%, representing a clear and often life-saving benefit. By contrast, in late preterm infants (34–36 weeks), the main advantage is reduced respiratory complications (about 4%), with little to no mortality reduction [40]. In the near term, respiratory risks decline further, yet theoretical concerns about neurodevelopmental vulnerability remain. In this setting, the precautionary principle supports limiting exposure where benefits are marginal, while a utilitarian perspective prioritises immediate survival gains despite uncertain long-term risks [74].

Given the ongoing debate about potential neurodevelopmental sequelae, some experts advocate for structured follow-up protocols for ACS-exposed children to monitor growth, cognition, and behaviour [75]. A study showed that hospitals in 5 major US states (California, Florida, Illinois, New York, Texas) are implementing the monitoring of ACS use in hospitals to ensure their most efficient usage [76]. However, in low-income countries (LICs), the guidelines on ACS use and their follow-ups are lacking, as shown by a WHO-led analysis of the ACTION-I trial. The trial also revealed that pregnant women in LICs are often unable to properly complete their course of medications due to entering labour before the scheduled time [77]. These disparities underscore the need for equitable global policies that integrate ACS exposure tracking with long-term neurodevelopmental monitoring, ideally embedded within national health systems alongside immunisation or growth surveillance programs.

Ultimately, the ethical and public health implications of ACS are related to the trade-off between well-established immediate survival benefits and uncertain long-term developmental risks. Communication, patient autonomy, and context-dependent health policy are important considerations. The development of standardised follow-up procedures for children exposed to ACS may help ensure that the well-established survival benefits of ACS are reflected in long-term health outcomes.

## 9. Clinical implications

ACS remain a cornerstone in perinatal care due to their well-established benefits. Evidence shows that ACS significantly reduces RDS, early neonatal mortality [78], and intraventricular haemorrhage in preterm infants as compared to women who had received expectant management [79]. These effects are particularly pronounced when ACS are administered within the optimal therapeutic window 24 hours to 7 days before delivery [80]. This administration was linked to a considerably higher survival rate as compared to unexposed infants and those who received ACS with shorter or longer intervals between administration and birth [81]. Such findings have made ACS a standard of care for women at risk of preterm birth. Despite these short-term benefits, concerns about long-term safety persist.

Some studies report that infants exposed to ACS undergo more frequent neurodevelopmental assessments compared with unexposed peers, although this may reflect heightened clinical vigilance rather than proven adverse outcomes [82]. Other investigations suggest that repeated ACS courses may be associated with lower birth weight, length, and head circumference, though findings are inconsistent across studies [83]. Maternal and fetal characteristics, along with the healthcare setting, can also affect the risk-benefit balance for ACS [17]. An increased number of days in the neonatal intensive care unit was found when the ACS to delivery interval was more than seven days [84]. The underlying biological pathways leading to preterm birth are complex and incompletely understood, further complicating predictions of ACS efficacy.

For accurate prediction of preterm birth, it is essential since the benefits of ACS are time-dependent. A single course of ACS will be most effective if the birth takes place within seven days. However, there are instances when a woman may undergo ACS and subsequently give birth at term. This discrepancy has led to concerns about potential unnecessary use, especially when clinicians take a liberal approach to administration [85].

Weighing the benefits of neonatal survival with potential long-term hazards remains an important consideration in obstetric decision-making. New evidence points to nuances in certain patient groups. In twin pregnancies, the benefits of ACS seem less pronounced than in singletons [74]. In mothers with co-morbidities such as diabetes and intrahepatic cholestasis of pregnancy, there are additional hazards and altered pharmacological profiles of ACS [86]. Elective caesarean delivery at late preterm or early term gestation is another area of uncertainty, with variable recommendations in guidelines.

The WHO currently recommends ACS primarily for women at high risk of imminent preterm birth under close medical supervision, underscoring the need for judicious patient selection [75]. Raising awareness of uncertainties regarding long-term outcomes, together with improving tools to predict preterm birth, may help clinicians optimise ACS use in borderline cases. Further research is needed to evaluate different dosing regimens, long-term effects in subgroups such as multiple pregnancies, and outcomes in high-risk obstetric contexts [78].

This review synthesises both mechanistic and epidemiologic data, carefully interpreting these streams of evidence to represent the current literature effectively. One of the strengths is the way in which we examined the multifactorial aetiology of ASD, including a wide range of potential genetic, environmental, and endocrine risk factors such as prenatal corticosteroid exposure, maternal stress, and the role of shared aetiologies such as SHANK2 and SHANK3 genes. By highlighting the overlap between neurodevelopmental and psychiatric trajectories (e.g., ADHD, depression) and by maintaining a cautious interpretation of findings—explicitly acknowledging that most studies did not directly assess ASD diagnoses—we enhance the credibility of our conclusions.

## 10. Limitations in existing research

Despite the considerable research that has been done on ACS, there are still certain limitations to the research that has been conducted so far. A study conducted through the help of a meta-analysis found that most studies had focused only on infants and toddlers, with few studies extending beyond that period of early childhood [23]. Another study found that only three studies had focused on the outcome in later childhood [14]. Without studies extending beyond that point, the actual prevalence and extent of the neurodevelopmental differences, including ASD, cannot be determined conclusively. In addition, certain confounding factors also need to be taken into consideration, such as the fact that in certain studies, pre-existing conditions in the mother and the fetus that were not related to the administration of GCs were not adequately differentiated from the effects of the corticosteroids themselves. For example, comparisons between exposed and unexposed groups often failed to adequately control for disease severity or obstetric risk, substantially reducing confidence in causal inferences [34].

Another important limitation is the absence of standardised instruments in the assessment of ASD. Various research works used different instruments in the assessment of the subject. General Conceptual Ability (GCA) was used to assess the neurodevelopmental outcomes of the subject [29]. The Social Communication Questionnaire (SCQ) was used in the direct screening of the subject's symptoms of ASD [36]. GCA is the cognitive ability of an individual to comprehend and solve complex problems, learn new things, and adjust to new conditions. It is a broad measure of the intellectual ability of a patient. Intellectual ability encompasses cognitive skills such as learning and reasoning. GCA is a measure of the intellectual ability of a person. GCA in the context of the subject's ASD is a complex condition. The study also used the Social Communication Questionnaire (SCQ) in the context of the subject's symptoms of ASD [36]. The SCQ is a screening tool used to assess symptoms associated with ASD. Although these tools offer valuable insights, heterogeneity in diagnostic approaches makes it difficult to compare results, which may lead to inconsistent findings.

Most importantly, none of the studies employed gold-standard diagnostic methods for ASD, which include the Autism Diagnostic Observation Schedule (ADOS-2), the Autism Diagnostic Interview-Revised (ADI-R), or clinical evaluations with reference to ICD-11/DSM-5 criteria. Without employing these diagnostic methods, it is difficult to ensure the accuracy of ASD risk estimates and may lead to potential misclassification bias. In fact, it is well known that without employing these diagnostic methods, it is not possible to ensure the accuracy of diagnostic findings for ASD. In summary, the combined limitations of short follow-up, residual confounding, and heterogeneous diagnostic criteria highlight the urgent need for long-term, methodologically rigorous studies employing standardised ASD assessment tools. Addressing these gaps is essential to clarify whether ACS exert any sustained neurodevelopmental effects and to guide future clinical and policy decisions.

## 11. Future directions

Further research is essential to clarify the potential long-term neurodevelopmental effects of antenatal corticosteroid (ACS) exposure. Well-controlled, prospective cohort studies are particularly needed to address limitations in existing literature, including residual confounding and short follow-up durations. To better elucidate specific risk patterns, future studies should examine outcomes by fetal sex, maternal inflammatory or autoimmune conditions, gestational age at exposure, steroid type and dosage, fetal growth status, and genetic susceptibility [34].

Multidisciplinary surveillance, involving close collaboration among various healthcare professionals, is crucial for improving patient outcomes and overall care quality. Collaboration among paediatrics, psychiatry, and obstetrics can ensure comprehensive assessment, planning, and management, while also avoiding duplication of services and optimising resource use. Such integrative care not only reduces costs but also enhances early risk identification and complication prevention, ultimately improving long-term neurodevelopmental outcomes.

## 12. Conclusion

Antenatal corticosteroids (ACS) remain a critical intervention for improving neonatal outcomes, particularly in preterm infants. While evidence hints at possible long-term neurodevelopmental effects, including ASD, current data remain limited, confounded, and inconclusive. The literature suggests that the timing, type, and dose of exposure, along with fetal and maternal factors, likely influence outcomes. Careful, long-term follow-up and high-quality prospective studies are needed to clarify risks, ideally integrating genetic, epigenetic, and standardised neurodevelopmental assessments. A multidisciplinary approach can help balance the immediate lifesaving benefits of ACS with potential long-term neurodevelopmental considerations.

## Abbreviations

Autism Spectrum Disorder (ASD)
Antenatal corticosteroids (ACS)
Glucocorticoids (GCs)
Corticosteroids (CS)
Respiratory Distress Syndrome (RDS)
Confidence Interval (CI)
Glucocorticoid receptors (GRs)
Hypothalamic–pituitary–adrenal (HPA)
Adjusted hazard ratios (aHR)
